# Giant Paratesticular Liposarcoma: A Case Report and Brief Review of the Literature

**DOI:** 10.7759/cureus.111996

**Published:** 2026-07-03

**Authors:** Salapu Ramesh Babu, Manilal B, V Kishore Reddy Bojja, Narendra Hulikal, Yeswanth N

**Affiliations:** 1 Surgical Oncology, Sri Venkateswara Institute of Medical Sciences, Tirupati, IND

**Keywords:** inguinoscrotal, paratesticular sarcoma, paratesticular tumor, radical orchidectomy, scrotal mass, well-differentiated liposarcoma

## Abstract

Paratesticular tumors are rare neoplasms, with liposarcomas of the spermatic cord representing an uncommon malignant subtype. These tumors typically grow slowly and may attain a considerable size before diagnosis because of their indolent clinical course. We report the case of a 65-year-old male patient who presented with a progressively enlarging left-sided scrotal swelling of five years' duration. Based on clinical examination and ultrasonographic findings, a left paratesticular neoplasm was suspected. Contrast-enhanced computed tomography revealed a large heterogeneous fat-containing lesion encasing the spermatic cord. The patient underwent complete surgical excision with left radical orchidectomy. Histopathological features (mature adipocytes with fibrous septae and atypical stromal cells), supported by immunohistochemistry (MDM2 intense nuclear positivity), confirmed the diagnosis of well-differentiated liposarcoma. The postoperative course was uneventful, and no adjuvant treatment was administered; he was kept on regular follow-up. He remains free of local recurrence and distant metastasis at 24 months of follow-up. This case highlights the importance of considering paratesticular liposarcoma in the differential diagnosis, particularly in patients presenting with slowly progressive scrotal swellings. Early diagnosis and complete surgical excision with negative margins remain the cornerstone of management and are essential for achieving optimal disease control and favorable long-term outcomes.

## Introduction

Primary tumors of the spermatic cord are exceedingly rare, with an estimated incidence of approximately 0.3 cases per million population. Among these, liposarcoma represents the most common histological subtype, accounting for nearly 50% of cases. The mean age at presentation is around 60-65 years [1].

Paratesticular liposarcomas are particularly uncommon, with fewer than 200 cases reported in the literature. Tumors larger than 10 cm are rare and are often referred to as "giant" tumors [2,3]. These lesions typically present as slow-growing, painless masses, often leading to delayed diagnosis. Due to their rarity, there are no standardized treatment guidelines. However, complete surgical excision with negative margins is widely accepted as the primary treatment modality. Adjuvant therapy is generally reserved for selected cases, such as incomplete resection or recurrence.

In this article, we report a rare case of a giant well-differentiated liposarcoma of the spermatic cord, highlighting its clinical presentation, diagnostic approach, and management.

## Case presentation

A 65-year-old male patient presented with a slowly progressive scrotal swelling of five years' duration, which was associated with a dragging type of pain and occasional burning micturition. He was a known case of diabetes mellitus and hypertension, both well-controlled on medication.

On examination (as shown in Figure 1a-1b), a large left scrotal swelling measuring approximately 30×20 cm was noted. The left testis was not separately palpable, while the right testis was normal. The penis was buried, and the meatus was visible. There was no cough impulse, and it was possible to get above the swelling. The transillumination test was negative. The overlying skin was stretched with loss of rugosities and no palpable inguinal lymphadenopathy.

**Figure 1:**
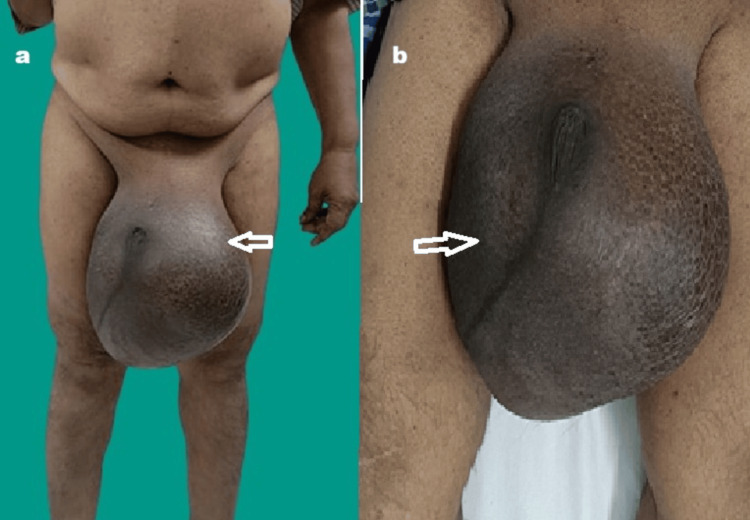
Clinical photographs demonstrating a scrotal swelling: (a) standing position (white arrows showing the scrotal swelling) and (b) supine position (white arrows showing the scrotal swelling)

Ultrasonography revealed a large heterogeneous scrotal mass with increased vascularity and no evidence of hydrocele. The left testis was not visualized separately. Contrast-enhanced computed tomography (as shown in Figure 2a-2d) demonstrated a large heterogeneous fat-density lesion measuring approximately 30×20×20 cm in the left hemiscrotum, with internal septations and soft tissue components. The lesion encased the spermatic cord and displaced the testis inferiorly with minimal free fluid noted within the tunica vaginalis. These features were suggestive of a paratesticular tumor, most likely liposarcoma of the left spermatic cord.

**Figure 2:**
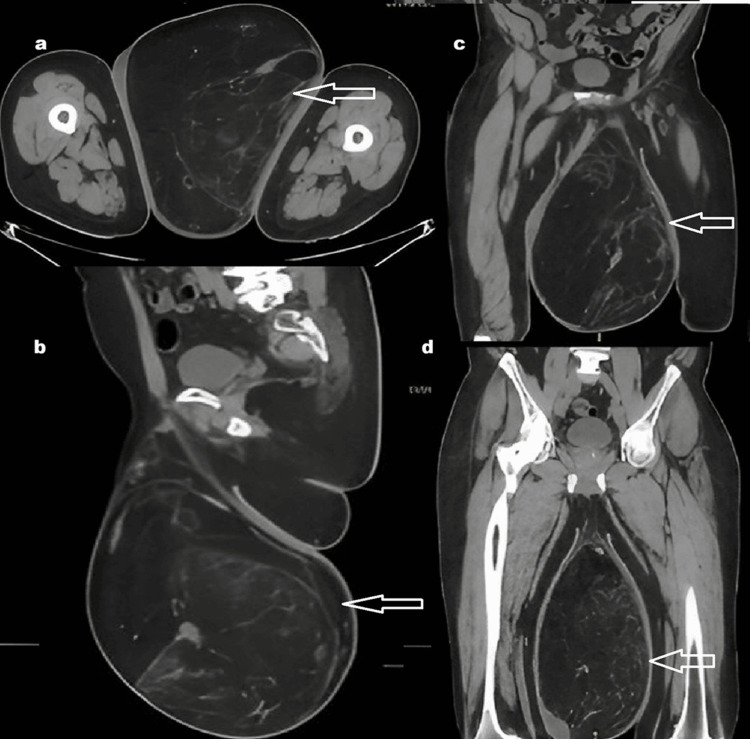
Contrast-enhanced computed tomography image of the patient showing a large heterogeneous fat-containing lesion (white arrow) in the left hemiscrotum: (a) axial view, (b) sagittal view, and (c-d) coronal view

The patient underwent surgical exploration. Intraoperatively, a 30×20×20 cm large solid mass was noted encasing the left spermatic cord. The left testis was identified and was densely adhered to the mass. Complete excision of the mass, along with left radical orchidectomy, was performed.

Gross examination revealed a well-circumscribed, multilobulated mass with a grey-yellow cut surface and focal myxoid areas without any hemorrhage or necrosis. Microscopy (as shown in Figure 3) showed a mature adipose tissue with fibrous septae and scattered atypical stromal cells. The left testis was normal with intact tunica vaginalis. Immunohistochemistry (as shown in Figure 4a) demonstrated strong nuclear positivity for MDM2, confirming well-differentiated liposarcoma.

**Figure 3:**
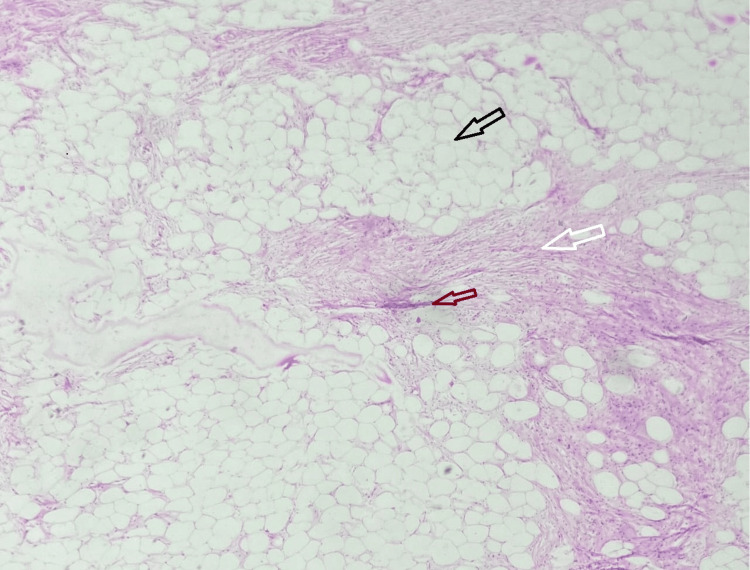
Histopathological image of the patient showing lobules of mature adipocytes (black arrow) with fibrous septae (white arrow) and atypical stromal cells (brown arrow), consistent with well-differentiated liposarcoma

**Figure 4:**
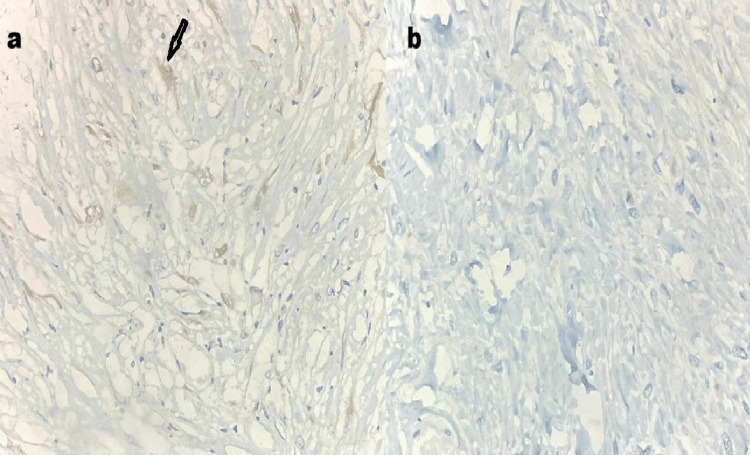
Immunohistochemistry demonstrating nuclear positivity for MDM2 and S100: (a) MDM2 (black arrow showing a strong nuclear positivity) and (b) S100

The perioperative course was uneventful, and the patient was discharged in a stable state. The case was discussed in the institutional tumor board, and no adjuvant treatment was recommended. He was kept on regular three-monthly follow-ups. He showed no signs of locoregional recurrence during the last visit, both clinically and radiologically (ultrasonography) at 24 months (as shown in Figure 5).

**Figure 5:**
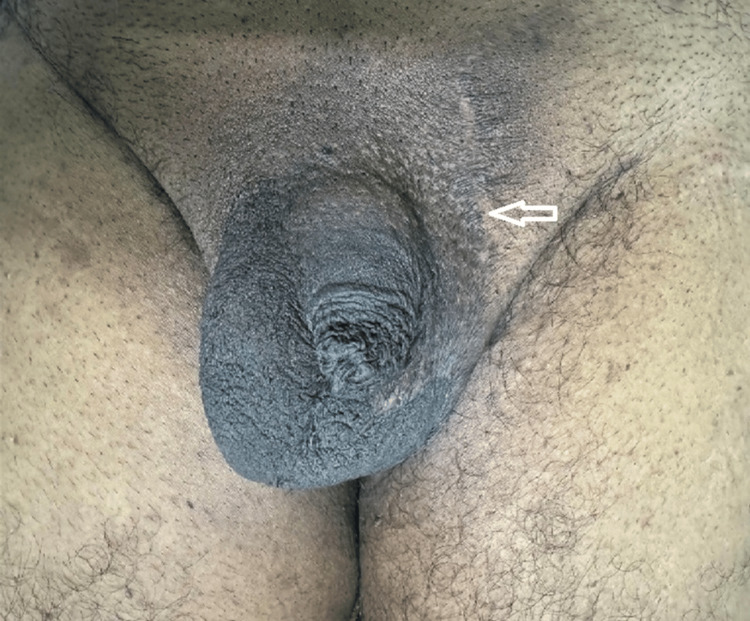
Postoperative follow-up image of the patient (white arrow showing the healed scar) at 24 months

## Discussion

Liposarcomas are malignant mesenchymal tumors arising from the adipocytic tissue and represent one of the most common soft tissue sarcomas. According to the 2020 World Health Organization Classification of Soft Tissue Tumours, soft tissue tumors are categorized into well-differentiated/atypical lipomatous, myxoid, dedifferentiated, pleomorphic, and myxoid pleomorphic subtypes [4].

Well-differentiated liposarcoma is a subset of liposarcomas that are composed of mature adipocytes and stromal cells with at least focal cytologic atypia. The variation in the terminology is based on the tumor location and risk of recurrence. For peripheral tumors that have a low risk of recurrence, the term atypical lipomatous tumor (ALT) is preferred. The term well-differentiated liposarcoma is used to denote deep-seated tumors (retroperitoneum, mediastinum, spermatic cord) which have a higher risk of recurrence and dedifferentiation [5].

Although termed as liposarcoma, the spermatic cord liposarcomas have an indolent course and an excellent prognosis. The first case of spermatic cord sarcoma was reported by Lesauvage in 1845 [6].

Although their pathogenesis is still unclear, they do not develop from the malignant transformation of an existing lipoma; rather, they arise de novo in the connective tissue surrounding the testis, epididymis, and spermatic cord. Molecularly, amplification of chromosome 12q13-15, particularly involving MDM2 and CDK4 genes, is characteristic [5].

Radiological imaging plays a key role in diagnosis. Ultrasonography can pick up the fatty content, but it can't differentiate between a lipoma and a liposarcoma. Cross-sectional imaging, such as contrast-enhanced computed tomography or magnetic resonance imaging, helps in the better characterization of fat content and tumor extent [7].

Complete surgical excision with negative margins is the standard of care and is the single most important prognostic factor in preventing recurrences. A retrospective study that included 25 patients with paratesticular liposarcomas concluded that the three-year recurrence-free survival rate was 100% and 29% in patients with negative and positive margins, respectively, highlighting the importance of complete surgical resection [8].

Lymphatic spread is uncommon; therefore, routine lymph node dissection is not recommended [9].

Due to the rarity of this tumor, the role of adjuvant therapy remains unclear. Chemotherapy and radiotherapy are generally reserved for recurrent, high-grade, or incompletely resected tumors. Vincristine, cyclophosphamide, and doxorubicin-based chemotherapy is used for high-grade and metastatic paratesticular liposarcomas [10]. 

Similar to previously reported cases, our patient presented with a slowly progressive scrotal mass and nonspecific clinical features that closely mimic benign scrotal conditions, often posing diagnostic challenges and delaying definitive diagnosis [10].

The differential diagnosis of paratesticular tumors is broad and includes benign conditions such as inguinal hernia, hydrocele, spermatocele, varicocele, epididymal cysts, post-traumatic hematoma, adenomatoid tumors, hemangioma, and leiomyoma, as well as malignant lesions including lymphoma, primary testicular neoplasms, and metastatic tumors [11,12]. 

Histopathological examination with immunohistochemistry confirms the diagnosis of well-differentiated liposarcoma, the most commonly reported histological subtype at this site. Furthermore, complete tumor excision with histopathologically negative margins was achieved, in accordance with current evidence emphasizing R0 resection as the cornerstone of management and a key determinant in preventing local recurrence. Therefore, this case supports and further validates the clinical and therapeutic observations described in earlier studies.

## Conclusions

Paratesticular liposarcoma is a rare entity that should be considered in the differential diagnosis of scrotal swellings. Preoperative diagnosis can be challenging due to nonspecific clinical features. Histopathological examination with immunohistochemistry is essential for definitive diagnosis. Complete surgical excision with negative margins remains the cornerstone of management and is critical in preventing recurrence. Long-term follow-up is essential due to the risk of local recurrence. Routine adjuvant chemotherapy or radiotherapy is not recommended and is reserved for cases with incomplete resection or recurrence.
